# Understanding Pain and Trauma Symptoms in Veterans From Resting-State Connectivity: Unsupervised Modeling

**DOI:** 10.3389/fpain.2022.871961

**Published:** 2022-05-10

**Authors:** Irina A. Strigo, Andrea D. Spadoni, Alan N. Simmons

**Affiliations:** ^1^Emotion and Pain Laboratory, San Francisco Veterans Affairs Health Care Center, San Francisco, CA, United States; ^2^Department of Psychiatry, University of California, San Francisco, San Francisco, CA, United States; ^3^Stress and Neuroimaging Laboratory, San Diego Veterans Affairs Health Care Center, San Francisco, CA, United States; ^4^Center of Excellence in Stress and Mental Health, San Diego Veterans Affairs Health Care Center, San Diego, CA, United States; ^5^Department of Psychiatry, University of California, San Diego, San Diego, CA, United States

**Keywords:** insula, nucleus accumbens, effective connectivity, neuroimaging, veterans, catastrophizing, chronic back pain, PTSD

## Abstract

Trauma and posttraumatic stress are highly comorbid with chronic pain and are often antecedents to developing chronic pain conditions. Pain and trauma are associated with greater utilization of medical services, greater use of psychiatric medication, and increased total cost of treatment. Despite the high overlap in the clinic, the neural mechanisms of pain and trauma are often studied separately. In this study, resting-state functional magnetic resonance imaging (rs-fMRI) scans were completed among a diagnostically heterogeneous sample of veterans with a range of back pain and trauma symptoms. Using Group Iterative Multiple Model Estimation (GIMME), an effective functional connectivity analysis, we explored an unsupervised model deriving subgroups based on path similarity in *a priori* defined regions of interest (ROIs) from brain regions implicated in the experience of pain and trauma. Three subgroups were identified by patterns in functional connection and differed significantly on several psychological measures despite similar demographic and diagnostic characteristics. The first subgroup was highly connected overall, was characterized by functional connectivity from the nucleus accumbens (NAc), the anterior cingulate cortex (ACC), and the posterior cingulate cortex (PCC) to the insula and scored low on pain and trauma symptoms. The second subgroup did not significantly differ from the first subgroup on pain and trauma measures but was characterized by functional connectivity from the ACC and NAc to the thalamus and from ACC to PCC. The third subgroup was characterized by functional connectivity from the thalamus and PCC to NAc and scored high on pain and trauma symptoms. Our results suggest that, despite demographic and diagnostic similarities, there may be neurobiologically dissociable biotypes with different mechanisms for managing pain and trauma. These findings may have implications for the determination of appropriate biotype-specific interventions that target these neurological systems.

## Introduction

Pain and trauma are highly comorbid conditions (1–6), especially among the military and veteran population (7, 8), and both are associated with dramatic changes in the brain structure and function (9, 10). Using experimental pain and task-based functional magnetic resonance imaging (fMRI), we and others have repeatedly shown that men and women with trauma demonstrate dysregulated behavioral (11, 12) and brain response to pain, depicted in the increased pain avoidance and dysregulated modulatory response within interoceptive, reward, and frontal modulatory networks (13–17).

Resting-state fMRI (rs-fMRI) allows for the identification of resting-state networks, which represent spatial distributions of synchronized fluctuations in blood oxygen level-dependent (BOLD) fMRI responses over time (18). Several neuroimaging studies have shown that chronic low back pain is associated with alterations in resting-state brain activity and dynamics (19–23). Changes within default mode network (DMN), salience network (SN), and cortico-striatal connectivity have all been reported (24, 25), and reduced resting-state insula connectivity in chronic back pain resolved following successful spinal surgery or zygapophysial (facet) joint block (26). Similarly, growing research demonstrates that chronic trauma is associated with alterations in resting-state connectivity such as DMN, SN, and central executive network (CEN, also referred to as the frontoparietal control network) (27, 28). Substantial overlap between changes in resting-state functional connectivity in both chronic pain and trauma is consistent with clinical observations of comorbidity between these conditions, and theoretical models wasproposed to underlie pain–trauma overlap (6). Nevertheless, to the best of our knowledge, no study has examined resting-state networks in individuals with comorbid pain and trauma symptoms. Understanding shared vs. unique underlying neural networks related to pain and/or trauma may provide the basis for novel, targeted interventions.

To examine the degree to which pain and/or trauma symptoms alter resting-state functional connectivity, we recruited a diagnostically heterogeneous sample of veterans with varying degrees of back pain and trauma symptoms and employed a model-free analysis approach with the goal of identifying mechanistically meaningful subgroups. Such diagnosis-free approach supports the National Institute of Mental Health (NIMH) Research Domain Criteria (RDoC) goals to understand mechanisms of psychiatric disorders and their comorbidities in a transdiagnostic manner (29). Data-driven approaches that characterize resting-state functional connectivity patterns show great promise in elucidating neurobiological mechanisms underlying clinical conditions and heterogeneous symptoms (30, 31). Especially, employing effective connectivity models, rather than Pearson's correlation and distance measures, can lead to an improved understanding of directional and causal relationships between networks rather than finding simple correlational associations (32, 33). A novel data-driven strategy called group iterative multiple model estimation (GIMME) has been shown to be particularly effective in deriving directed pathways and is successful in separating clinically heterogeneous population (34, 35). GIMME is a version of unified structural equation modeling (uSEM) that uses time-series analysis within an SEM framework to estimate the prediction strength of directed paths between variables controlling for lagged and contemporaneous effects of all the variables entered into the model (36). Simulation and experimental evidence suggest that GIMME is robust against problems associated with signal-to-noise ratio, hemodynamic response variability, and low sample sizes (35, 37). A modification of GIMME, called subgrouping within GIMME (or “S-GIMME”), can identify neural subtypes in small sample sizes and outperforms other clustering methods (34).

The current study sought to utilize S-GIMME with resting-state functional connectivity to identify whether pain and trauma symptoms are associated with differential functional connectivity patterns at rest in a diagnostically heterogeneous sample of veterans within brain regions implicated in the experience of pain and trauma [i.e., insula, anterior cingulate cortex (ACC), thalamus, posterior cingulate cortex (PCC), and nucleus accumbens (NAc)]. We hypothesized that a model-free approach would identify specific connectivity patterns for increased pain, trauma, and their overlap.

## Materials and Methods

Fifty-seven veterans (9 women, mean ± SD age: 35.5 ± 3.75, range 28–44) gave written informed consent to participate in this study, which was approved by the University of California San Francisco Human Research Protection Program and Veterans Affairs San Francisco Healthcare System Research and Development Committee. Participants were recruited from the VA San Francisco Healthcare System (VASFHS) *via* advertisement materials (e.g., flyers posted in the hospital public areas) or through referrals from other ongoing research projects at this site and through mining VA electronic health records. The sample included individuals with a range of back pain and/or trauma symptoms. Study inclusions were (1) veterans between the ages of 18 and 55; (2) veterans experiencing back pain and/or history of life-threatening trauma during combat and experiencing distress, such as excessive anxiety, jumpiness, or nightmares, related to trauma; (3) veterans that were able to undergo MRI (e.g., have no contraindications for MR); and (4) veterans currently not taking narcotic medication. Subjects were excluded from the study if they: (1) had a current or lifetime history of bipolar disorder, psychosis, eating disorders, and obsessive-compulsive disorder (OCD); (2) had a history of alcohol and/or drug dependence within 3 months of study participation; (3) showed a neurological disorder that might be associated with cognitive dysfunction (including head trauma associated with fracture, pre-deployment traumatic brain injury (TBI) resulting in loss or alteration of consciousness, cerebrovascular accident, or intracranial surgery, aneurysm, and seizures); (4) had prior neurosurgery; (5) had irremovable ferromagnetic material; (6) were pregnant; (7) were claustrophobic; (8) had TBI with loss of consciousness (LOC) > 10 min; and (9) had vision problems uncorrectable with lenses. Three subjects were excluded from the analyses due to extensive motion in the MRI scanner.

### Clinical Measures

All subjects underwent a Structural Clinical Interview administered by trained interviewers according to the Mini-International Neuropsychiatric Interview (38) to establish current and past psychiatric diagnoses. In addition, each subject completed a Research Electronic Data Capture (REDCap) battery of questionnaires assessing specific pain, trauma, and comorbid symptoms. The battery included pain severity and interference from the Brief Pain Inventory (BPI) (39) and post-traumatic stress disorder (PTSD) checklist for DSM5 (PTSD Checklist version 5, PCL-5) (40) to assess pain and trauma symptoms, respectively. In addition, subjects completed Beck Depression Inventory-2 (BDI-2) (41) to assess the severity of depressive symptoms, the Pain Catastrophizing Scale (PCS) (42), and a short form of the Pain Anxiety Symptoms Scale (PASS-20) (43) to assess pain-related cognitions, and the Combat Exposure Scale (CES) (44) to access wartime stressors experienced by combatants. Note that we used the symptoms spectrum approach in the current study; nevertheless, each subject was also given a tentative group assignment based on meeting/not-meeting trauma criterion A, and/or chronic pain diagnosis, as recommended (45, 46).

### MRI Acquisition and Preprocessing

Brain imaging data were acquired on 3-T Siemens Skyra MRI scanner. All subjects underwent (1) a high-resolution T1-weighted structural scan (320 slices; repetition time, TR = 2,400 ms; echo time, TE = 2.24 ms; flip angle, FA = 8°; field of view, FOV = 166.4 mm × 240 mm; matrix size = 208 × 300; voxel size = 0.8 mm × 0.8 mm × 0.8 mm); and (2) rs-fMRI scan (66 slices in interleaved ascending order; TR = 1,000 ms; TE = 35 ms; FA = 62°; FOV = 208 mm × 208 mm; matrix size = 104 × 104; voxel size = 2 mm × 2 mm × 2 mm; MB factor = 6) while laying comfortably in the MRI scanner. The rs-fMRI run was 8:22 min in length, during which 502 functional volumes were acquired. Data were preprocessed using fMRIPrep 1.5.7 (47), which is based on Nipype 1.4.0 (48, 49) and followed the recommended pipeline. Briefly, data were (1) de-spiked by removal of regional statistical outliers with interpolated regional means, (2) temporarily corrected to slice acquisition, (3) spatially corrected for six direction motion parameters (*x, y, z*, roll, pitch, and yaw) and their derivatives, (4) aligned to anatomical T1 and normalized brain space, and (5) scaled for percent signal change. For more details of the pipeline, see the section corresponding to workflows in fMRIPrep's documentation (47). Data were smoothed with a Gaussian kernel (full-width half-maximum, FWHM = 5 mm) and band-pass filtered (range [0.008, 0.09] Hz).

### Resting-State Networks

**Figure 1:**
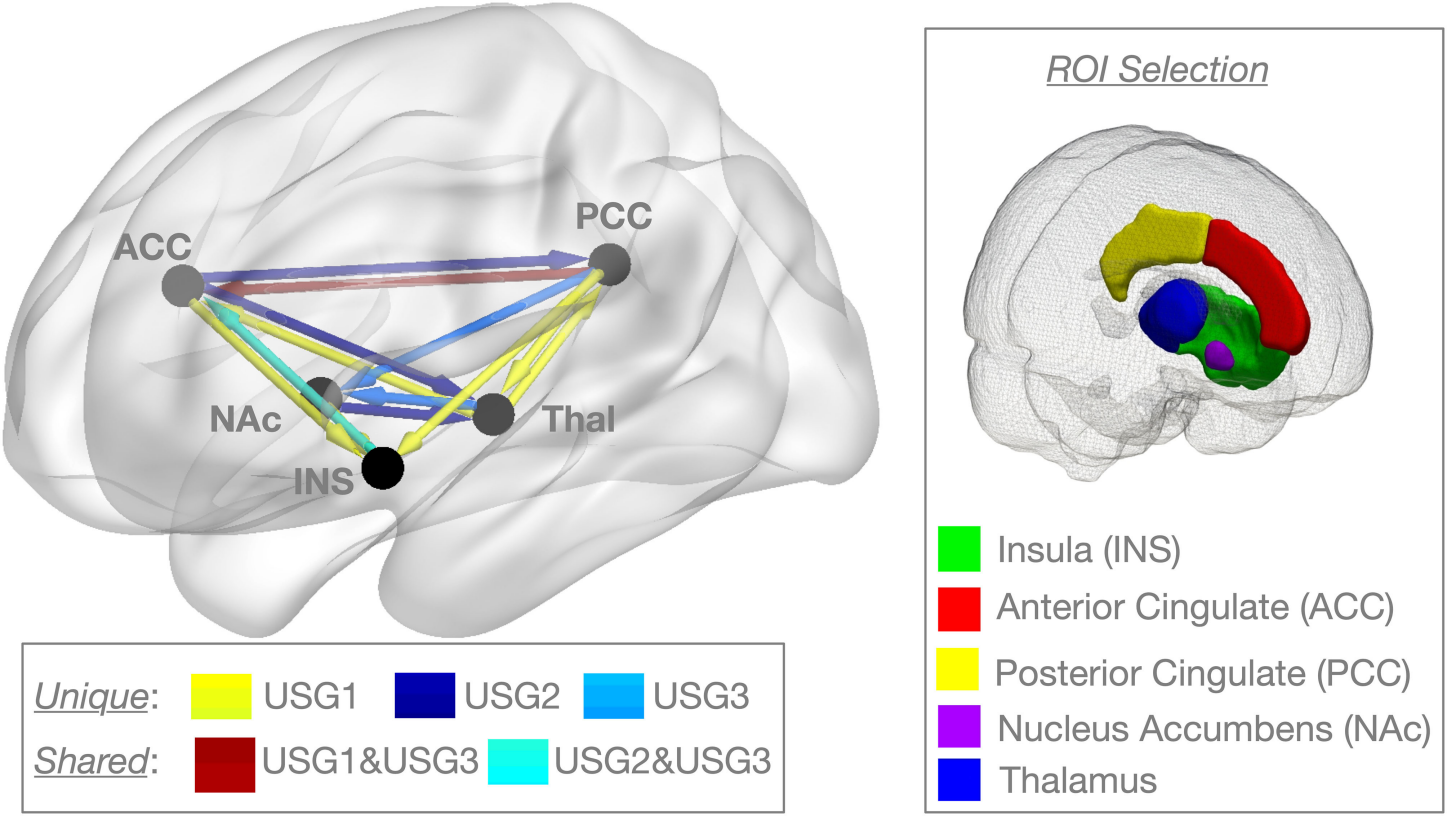
Unsupervised GIMME (USG) identified common and unique effective connectivity for the three subgroups based on effective connectivity paths. Subgroup 1 [USG1, yellow, *n* = 16, 7 connections] was characterized by functional connectivity to the insula from NAc, ACC, and PCC, and from the thalamus to ACC and bidirectional interconnections between the thalamus and PCC. Subgroup 2 [USG2, indigo, *n* = 23, 4 connections] was characterized by functional connectivity from the ACC and NAc to the thalamus and from ACC to PCC. Subgroup 3 [USG3, sky blue, *n* = 15, 4 connections] was characterized by functional connectivity from the thalamus and PCC to NAc. Both USG1 and USG3 had overlapping functional connections from PCC to ACC (garnet), whereas USG2 and USG3 were characterized by overlapping functional connectivity from the insula to ACC (cyan). No connections were shared between USG1 and USG2. (Right) The box on the right shows pictorial of the five regions of interest (ROIs) extents of voxels included in signal averages [Hammers_mith atlas n30r83 (51)]. We extracted the mean BOLD time series from the average activity across voxels using five bilateral regions averaged across both sites; only one side is displayed for clarity. IC, insular cortex; ACC, anterior cingulate cortex; Thal, thalamus; PCC, posterior cingulate cortex; NAc, nucleus accumbens; BOLD, blood oxygen level-dependent. c.f. text for details.

We extracted the mean BOLD time series from the average activity across voxels using five bilateral regions of interest (ROIs) averaged across both sites: thalamus, insula, ACC (SN and interoception), PCC (DMN), and NAc (reward/relief) using Hammers' atlas (50, 51) (see inset on the right in Figure 1). The mask was chosen based on the fact that these regions are part of the networks that are heavily implicated in both pain and trauma (52–55). Considering that our sample was heterogeneous, which is typical for the veteran population, identifying symptom-specific vs. comorbid and diagnostic vs. symptomatic changes in functional connectivity between these regions at rest can provide valuable insight into the biological underpinnings of pain and/or trauma symptoms, diagnoses, and potential treatment targets. These five ROIs were then entered into connectivity classification analyses with unsupervised S-GIMME (see below) to derive subgroups based on path similarity.

### Unsupervised S-GIMME

An unsupervised subgroup classification was performed using unsupervised subgrouping-GIMME, or S-GIMME, in *R* (R Core Team) in order to identify whether clinically relevant subgroups of subjects can be derived based upon resting-state effective connectivity patterns (34). Unsupervised S-GIMME can be used to not only identify common pathways across all subjects but also importantly classify subjects within subgroups based upon unique connectivity profiles and estimate subject-level connectivity (34). First, unsupervised S-GIMME searches across individuals to derive a connectivity profile of lagged (lag = 1 TR) and contemporaneous directed pathways that are common for the majority of subjects (group-level) and prunes the remaining paths. Furthermore, unsupervised S-GIMME uses unsupervised community detection [Walktrap; (56, 57)] on the similarity matrix derived from the individual-level estimates of the group-level connections to identify an optimal number of subgroups who have similar connectivity profiles. Once subgroups are identified, unsupervised S-GIMME searches for unique connectivity paths for each data-driven subgroup. Finally, unsupervised S-GIMME identifies individual-level connections using the group- and subgroup-derived temporal patterns as priority. After identification of subgroups derived from unsupervised S-GIMME, we compared subgroups on clinical measures to examine clinical relevance of the identified subgroups. Like previous reports (58, 59), we only report on contemporaneous pathways.

### Subgroup Characterization Analysis

Statistical analysis of clinical and psychological variables was conducted in R and JASP (60). JASP (Version 0.14.1), ANCOVAs, *t*-tests, and chi-square tests were used to compare S-GIMME subgroups on clinical and demographic variables (i.e., age and education). For pain and trauma symptoms, results were uncorrected for multiple comparisons due to *a priori* assumption. Other results were corrected for multiple comparisons using Dunn's method. *Post-hoc* analyses also explored associations of unique connectivity by subgroup with clinical variables using Spearman's correlations.

## Results

### Unsupervised S-GIMME

Unsupervised S-GIMME produced a three-group solution (Figure 1), sorting 16 subjects into subgroup 1 (USG1, yellow), 23 subjects into subgroup 2 (USG2, indigo), and 15 subjects into subgroup 3 (USG3, sky blue). The connectivity and directionality of connections between ROIs within each subgroup are illustrated in Figure 1 (left). Overall, USG1 had 7 connections between 5 ROIs, whereas USG2 and USG3 had 4 connections. USG1 was characterized by functional connectivity to the insula from NAc, ACC, and PCC and from the thalamus to ACC and bidirectional interconnections between the thalamus and PCC. USG2 was characterized by functional connectivity from the ACC and NAc to the thalamus and from ACC to PCC. USG3 was characterized by functional connectivity from thalamus and PCC to NAc. Several connections were overlapping between the subgroups. Both USG2 and USG3 were characterized by overlapping functional connectivity from the insula to ACC, whereas USG1 and USG3 had overlapping functional connections from PCC to ACC. No connections were shared between USG1 and USG2. We have displayed (Figure 1, right) the extent of voxels included in signal averages for each of the five ROIs in Figure 1 [Hammers_mith atlas n30r83 (51)].

### Demographic, Clinical, and Psychological Variables

Unsupervised S-GIMME subgroups did not differ significantly on age [*F*_(2,1.50)_ = 1.41, *p* = 0.254], sex (χ2 = 1.972, *p* = 0.373), race/ethnicity (χ2 = 8.295, *p* = 0.405), education level [*F*_(2,1.48)_ = 0.766, *p* = 0.471], marital (χ2 = 8.255, *p* = 0.409), or employment (χ2 = 0.609, *p* = 0.996) status (see Table 1). Similarly, there was no significant difference in the reported mild TBI (χ2 = 0.414, *p* = 0.813) or the proportion of veterans meeting/not-meeting trauma and/or chronic pain criteria (χ2 = 6.208, *p* = 0.400, **Figure 3**). Similarly, no significant differences between subgroup analyses in combat exposure were observed [see Table 1, *F*_(2,1.50)_ = 0.347, *p* = 0.708].

**Table 1:** Subjects' characteristics.

	**USG1**	**USG2**	**USG3**	**Fstat/ χ2**	***p*-value**
N	16	23	15		
*Age*	34.44 (4.15)	35.7 (3.43)	36.4(3.76)	1.41	n.s.
*Sex, number of subjects (percent)*				1.972	n.s.
Male	12(75%)	21(91%)	12(80%)	
Female	4(25%)	2(9%)	3(20%)		
*Ethnicity/Race*				8.295	n.s.
Caucasian	6(38%)	15(68%)	8(57%)		
African-American	1(6%)	0(0%)	1(8%)		
Asian/Pacific Islander	3(19%)	0(0%)	1(7%)		
Hispanic/Latino	2(13%)	4(18%)	2(14%)		
Mixed	4(25%)	3(14%)	2(14%)		
*Education*	14.5(1.16)	14.91(1.02)	15.143(1.70)	0.766	n.s.
*Marital Status, number of subjects (percent)*				8.255	n.s.
Single	1(6%)	6(26%)	5(33%)		
In a relationship	5(31%)	9(39%)	5(33%)		
Married	8(50%)	5(22%)	4(27%)		
Divorced/separated	2(13%)	2(9%)	0(0%)		
*Employment, number of subjects (percent)*				0.609	n.s.
Employed	12(75%)	16(69%)	10(67%)		
Out of work	1(6%)	2(9%)	2(13%)		
Student	2(13%)	3(13%)	2(13%)		
Other	1(6%)	2(9%)	1(7%)		
*Clinical and Psychological Variables*
*Mild TBI, number of subjects (percent)*				0.414	n.s.
Yes	9(56%)	13(57%)	7(47%)		
No	7(44%)	10(43%)	8(53%)		
*PTSD Checklist (PCL)*	9.81(9.16)	12.34(15.76)	24.53(20.11)	4.107	<0.05
*Pain Severity (BPI)*	2.05(2.26)	1.93(2.06)	3.44(2.42)	2.220	n.s.
*Pain Interference (BPI)*	1.59(2.31)	1.67(2.02)	3.02(2.24)	2.161	n.s.
*Beck Depression Inventory2 (BDI2)*	7.31(7.31)	12.17(11.59)	16.13(13.18)	2.815	0.069
*Pain Catastrophizing Scale (PCS)*	8.44(7.32)	9.61(9.99)	18.4(14.47)	3.963	<0.05
*Pain Anxiety Symptoms Scale (PASS)*	14.25(10.77)	17.39(15.79)	28.47(17.17)	4.421	<0.05
*Combat Exposure Scale (CES)*	12.25(10.83)	11.13(12.14)	13.27(12.87)	0.347	n.s.

*USG [1,2,3]– unsupervised GIMME subgroups based on effective connectivity path similarities; BPI – brief pain inventory; n.s. p > 0.05*.

**Figure 2:**
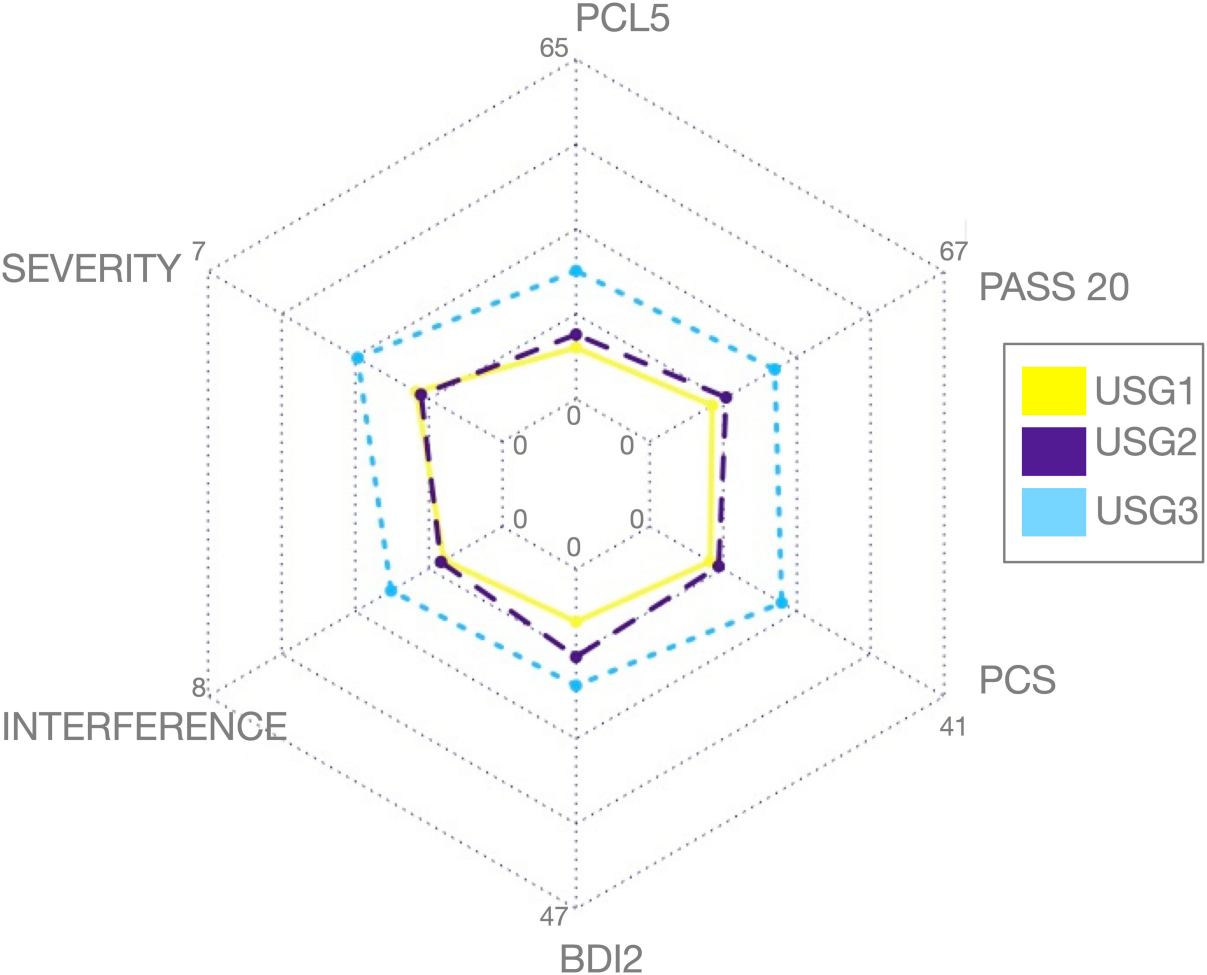
Graphic representation of clinical and psychological variables among the three subgroups identified by unsupervised S-GIMME based on effective connectivity paths at rest. Subgroup 1 [USG1, yellow, *n* = 16], subgroup 2 [USG2, indigo, *n* = 23], and subgroup 3 [USG3, sky blue, *n* = 15]. The radar plot is bounded by the minimum and maximum scores in the current sample for each scale (these averages for each subgroup are displayed for reference). c.f. Table 1 for statistical details. Severity, pain severity; Interference, pain interference; both from Brief Pain Inventory (BPI); PCS, Pain Catastrophizing Scale; PCL-5, PTSD Checklist version 5; BDI-2, Beck Depression Inventory-2; PASS-20, Pain Anxiety Symptoms Scale; c.f. text for details.

Conversely, unsupervised S-GIMME subgroups differed significantly on several clinical symptoms and psychological measures (see Table 1). This is graphically demonstrated by the radial plot in Figure 2.

**Figure 3:**
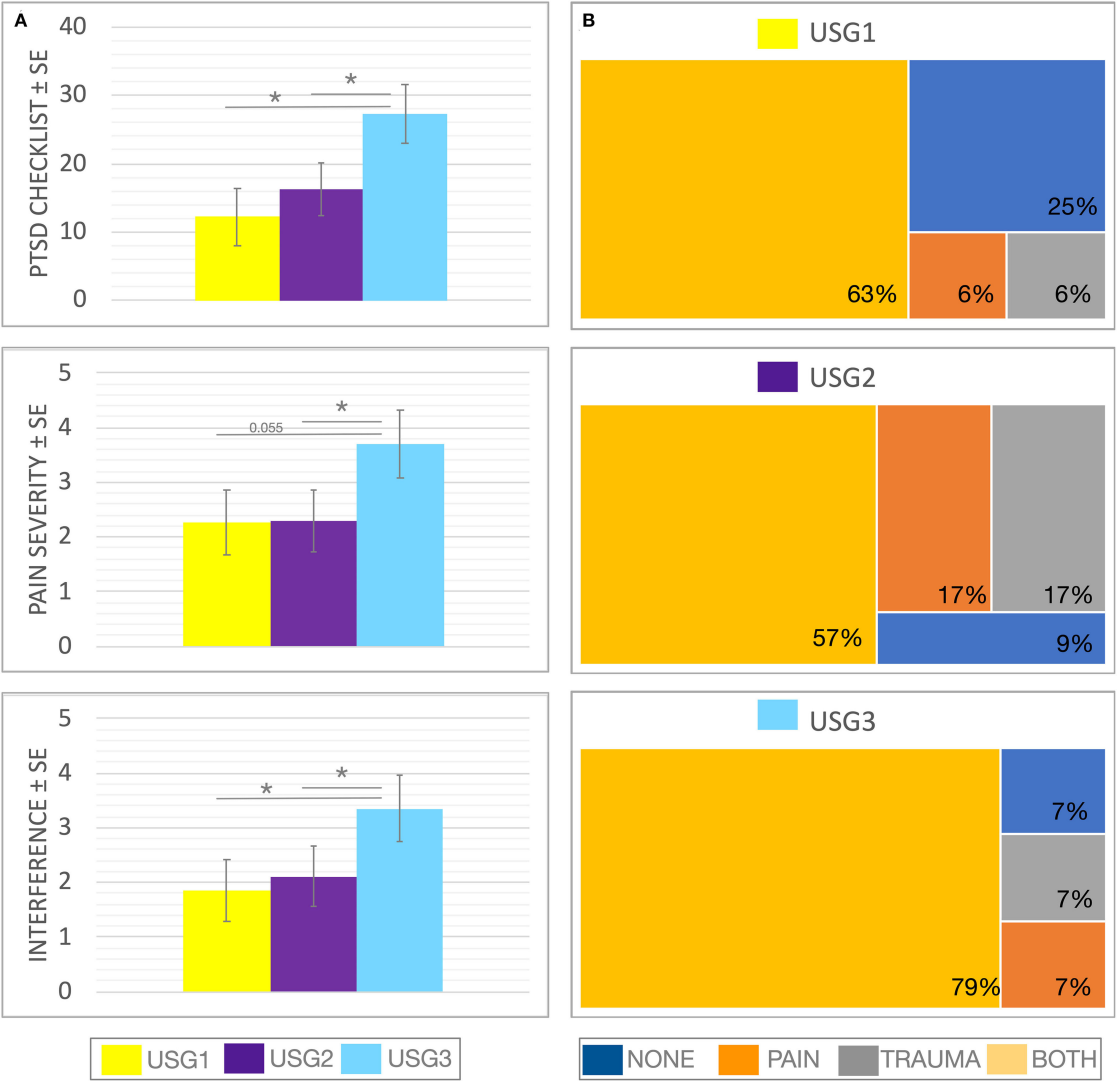
Clinical **(A)** and diagnostic **(B)** characteristics of GIMME subgroups identified by unsupervised S-GIMME based on effective connectivity paths at rest. **(A)** Box plots of trauma and pain symptoms (marginal mean ± SE) for each of the subgroups. (top) PCL scores were significantly different [*F*_(2,1.50)_ = 4.107, *p* < 0.05], ANCOVA (GIMME subgroup as a fixed factor and sex as covariate); Dunn's *post-hoc* comparisons indicated that the mean in the USG3 group was significantly higher than that in the USG1 and USG2 (*p*-values < 0.05), and there was no significant difference between USG1 and USG2 (*p* = 0.469). (middle) Pain severity (from BPI) showed a tendency for a significant GIMME subgroup effect while covarying for sex [*F*_(2,1.50)_ = 2.220, *p* = 0.1]; Dunn's *post-hoc* comparisons indicated that the mean in the USG3 group was significantly higher than that in the USG2 (*p* < 0.05) and approached significance when compared with USG1 (*p* = 0.055); there was no significant difference between USG1 and USG2 (*p*-values > 0.5) (bottom) Pain interference (from BPI) showed a tendency for significant GIMME subgroup effect while covarying for sex [*F*_(2,1.50)_ = 2.161, *p* = 0.1]. Dunn's *post-hoc* comparisons indicated that the mean in the USG3 group was significantly higher than that in the USG2 and USG1 (*p* < 0.05). There was no significant difference between USG1 and USG2 (*p*-values > 0.5). **(B)** Treemap charts show a hierarchical view of tentative diagnostic groupings within each of the GIMME subgroups. The symptoms-based approach was utilized in the current work; nevertheless, each subject was also given a tentative group assignment based on meeting/not-meeting trauma criterion A, and/or chronic pain diagnosis, as recommended (45, 46). NONE—no pain/no trauma, PAIN—pain/no trauma, TRAUMA—trauma/no pain, and BOTH—pain/trauma. The proportion of each diagnostic group within each GIMME subgroup is shown. There was no significant differences between subgroup analyses in the proportion of veterans meeting/not-meeting trauma and/or chronic pain criteria (χ2 = 6.208, *p* = 0.400); * <0.05, c.f. Table 1 and text for details.

As can be seen in Figure 2, USG1 was characterized by low scores of pain and psychological symptoms, USG3 was characterized by high scores of pain and psychological symptoms with USG2 being in the middle. GIMME subgroup differences for pain and trauma symptoms are shown in Figure 3A. As determined by one-way ANCOVA with GIMME subgroup as a fixed factor and sex as a covariate, PCL-5 scores were significantly different [*F*_(2,1.50)_ = 4.107, *p* = 0.022]. Dunn's *post-hoc* comparisons of the GIMME subgroups indicated that the mean in the USG3 group was significantly higher than that in the USG1 and USG2 (*p* < 0.05) groups, and there was no significant difference between USG1 and USG2 (*p* = 0.469). Both pain severity and pain interference showed similar differences, yet ANCOVA only showed a tendency for significant GIMME subgroup effect while co-varying for sex [pain severity: *F*_(2,1.50)_ = 2.220, *p* = 0.1; pain interference: *F*_(2,1.50)_ = 2.161, *p* = 0.1]. Since we hypothesized *a priori* that GIMME subgroups would differ in pain and/or trauma symptoms, we performed Dunn's *post-hoc* comparisons of the GIMME subgroups. *Post-hoc* analysis indicated that the mean in the USG3 group was significantly higher than that in the USG2 for pain intensity and pain interference (*p* < 0.05). Relative to USG1, USG3 was significantly higher on pain interference (*p* < 0.05) and approached significance on pain intensity (*p* = 0.055). There were no significant differences in either pain intensity or pain interference between USG1 and USG2 (*p*-values >0.5). When PCS, BDI-2, and PASS-20 scores were examined, the one-way ANCOVA with GIMME subgroup as a fixed factor and sex as a covariate showed that PCS scores, as well as the PASS-20 scores [_F(2, 1.50)_ = 4.421, *p* = 0.017], were significantly different [*F*_(2,1.50)_ = 3.963, *p* = 0.025], whereas BDI-2 scores approached significance [*F*_(2,1.50)_ = 2.815, *p* = 0.069] to be different between the three GIMME subgroups.

### Exploratory Correlational Analysis

**Figure 4:**
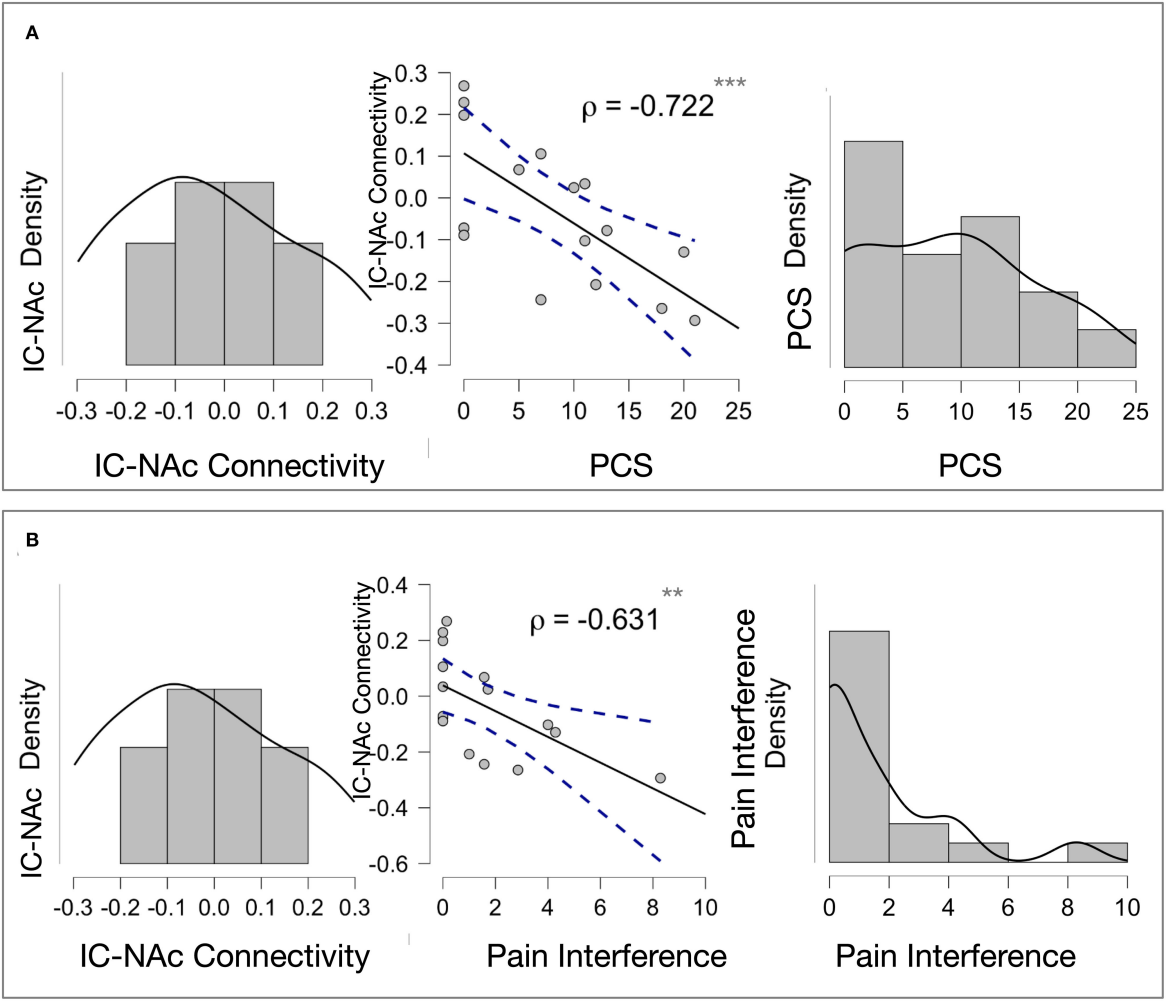
Exploratory correlations: **(A)** Insula—Accumbens Connectivity and Pain Catastrophizing Association in USG1. NAc connection to the insula, which was unique to subgroup USG1, or the group with the minimal levels of psychological and pain symptoms, was inversely and significantly correlated with pain catastrophizing scores (PCS) (ρ = −0.722, *p* < 0.001). The Scatter plot (middle) shows Pearson's correlations for a visual demonstration. Density plots for NAc to insula connectivity (left) and PCS scores (right) within USG1 are shown. **(B)** Insula—Accumbens and Pain Interference Association in USG1. NAc connection to the insula, which was unique to subgroup USG1, was inversely and significantly correlated with pain interference scores (ρ = -0.631, *p* = 0.004). The Scatter plot (middle) shows Pearson's correlations for a visual demonstration. Density plots for NAc to insula connectivity (left) and interference scores (right) are shown. No other subgroup-specific connections showed a significant relationship with the psychological and/or pain measures. ** <0.01, *** <0.001; PCS, Pain Catastrophizing Scale; Interference, pain interference measure from Brief Pain Inventory (BPI).

To examine which connections were indicative of a vulnerability or resiliency, GIMME subgroup-specific correlations were performed between pain and trauma scores and individual path estimates for subgroup-unique connections. For the USG1, or the group with the minimal levels of psychiatric and pain symptoms, we found that the NAc connection to the insula, which was unique to this group, was inversely correlated with pain interference (ρ = -0.631, *p* = 0.004) and PCS (ρ = −0.722, *p* < 0.001) scores (Figures 4A,B). No other subgroup-specific connections showed a significant relationship with the psychological and/or pain measures.

## Discussion

The present study reports new findings of unique functional connectivity patterns associated with pain and trauma symptoms during rest in a sample of veterans with a spectrum of pain and trauma symptoms. Using an unsupervised modeling approach, we identified unique connectivity patterns in these veterans at rest. Specifically, we found three separate subgroups showing different functional connectivity between the chosen ROIs that play an important role in pain, reward, and trauma (52–55), despite having similar demographic and clinical diagnostic characteristics. This study suggests that even though they may have similar demographic and diagnostic characteristics, there may be neurobiologically dissociable biotypes with different mechanisms for handling pain and trauma. This may have implications for the determination of appropriate biotype-specific interventions that target these neurological systems.

We found that the subgroups, defined by unsupervised S-GIMME as showing differential functional connectivity patterns at rest, were clearly separable in their clinical symptoms of trauma and pain. Specifically, we found that the group characterized by the lowest pain and trauma scores (USG1), or the least clinically impaired subgroup was highly connected overall, i.e., compared to the other two groups, USG1 showed more subgroup-unique connections, and overlapping functional connections with the other subgroups. This so-called “low symptom group” was characterized by functional connectivity from NAc, ACC, and PCC to the insula, and also from the thalamus to ACC and bidirectional interconnections between the thalamus and PCC. The insula plays a pivotal role in emotional and interoceptive processing (52, 61, 62). Besides its proposed role as an interoceptive sensory cortex (63, 64), substantial evidence supports the notion that the insula represents a hub for dynamic switching between different networks, i.e., based on the emotional and interoceptive needs of an organism, the insula helps to access attention, working memory, and other higher-order cognitive processes (62, 65). Not surprisingly, many pain and psychiatric disorders are thought to be related to faulty insula connectivity (66–71). Such interpretation is consistent with our findings of the insula being a hub for connectivity at rest in the “low symptom,” i.e., the least clinically impaired, subgroup. Interestingly, this “low symptom” subgroup is the only group that showed unique connectivity from NAc to the insula, which also was inversely correlated with pain interference and PCS scores in this subgroup. In other words, those with the lowest pain catastrophizing and pain interference scores had the highest functional connectivity from NAc to the insula, potentially suggesting the protective role of such connections. The NAc is at the core of the reward system, as it integrates emotional information to modulate motivated behavior (72, 73). In addition, a meta-analysis of healthy subjects has shown that spontaneous activity in the NAc predicts activity in the insula, suggesting consistent co-activation among these regions (74). Our study shows that co-activation of these regions plays an important role in discriminating between anticipating more painful vs. anticipating less painful experimental pain stimuli (75). This is consistent with the reported activation of NAc during pain predictive cues, the anticipation of pain relief, and the reported dopaminergic inputs to NAc during both reward and punishment (76–80). Furthermore, the motivation-decision model of pain states that actions are influenced by decisions about whether to approach something pleasant (a reward) or avoid something unpleasant (pain/loss) (53). We believe that NAc–insula connection provides a balance between pain and pain relief, and functional connection between these regions at rest thus underlies a healthy (or resilient) way to respond to stressful events such as trauma and/or pain.

With regard to the thalamus to ACC connectivity that was also unique in the “low symptom” subgroup, both the ACC and the thalamus are implicated in the affective/emotional aspects of pain and are thought to play important roles in pain modulation. More specifically, evidence suggests that, in response to pain, the ACC engages lower parts of the descending pain control system (i.e., PAG, hypothalamus, and rostral ventromedial medulla), which in turn exert an opioid-dependent inhibitory influence on spinal nociceptive processing, reducing nociceptive input to thalamic and cortical regions, and ultimately leading to a reduced pain experience (81). As such, it is possible that our observation of greater connectivity between the ACC and the thalamus in the “low symptom” subgroup could reflect the proper functioning of the descending analgesic pathway that is ready to engage in the setting of painful/traumatic experiences.

We found that the GIMME subgroup that demonstrated the highest pain and trauma symptoms, or the comorbid “high symptom” subgroup, reported the highest pain catastrophizing, pain anxiety, and depressive symptom severity scores and was characterized by functional connectivity from the thalamus to NAc and from PCC to NAc. Considering the above fact, the lack of insula connectivity with NAc and with other regions in this subgroup can potentially underlie the highest clinical impairment in this subgroup. The fact that NAc appears to be a hub for connectivity at rest in the “high symptom” subgroup may not be surprising, considering the role that NAc plays in chronification of pain in both human and animal models (25, 82–85). Furthermore, chronic pain patients suffer from anhedonia, decreased motivation, and exhibit impaired value-based decision-making—all properties of the cortico-striatal system (86–88). Not surprisingly, decreased motivation and impaired valence-based processing are typical for PTSD (17), and NAc circuitry is implicated in post-trauma active avoidance (89). The thalamus is a central hub of homeostatic information (90), whereas the PCC is implicated in regulating the focus of attention (e.g., internal vs. external) (91). Greater NAc connectivity between these regions in this “high symptom” subgroup may work jointly to influence pain catastrophizing and pain anxiety *via* the increased projection of internal pain states and the increased demand to direct internal attention away from such states.

We also found that “low symptom” and “high symptom” subgroups had overlapping functional connections from PCC to ACC. We speculated that increased connectivity here underlies coping with stress, yet in one group, it is an adaptive flexible response, such as mind wandering and cognitive control, whereas in another, it is maladaptive, conveying learned helplessness (92).

Our model-free approach identified another subgroup that was characterized by low pain and trauma symptoms, similar to the “low symptom” subgroup, but was functionally separated from the “low symptom” and “high symptoms” subgroups. This, so-called, “medium symptom” subgroup showed unique connections from ACC to PCC. PCC is a key part of DMN). More DMN connectivity in this group can likely be understood in the context of DMN regulatory response to pain, depression, and trauma. Evidence within the broader chronic pain literature suggests that stronger DMN connectivity may reflect an enhanced ability to engage in mind wandering (i.e., attention fluctuating away from the present sensory environment) (93, 94), thereby decreasing the salience of pain (95, 96). Therefore, it is possible that our analysis revealed a subgroup that is different from the “low symptom” subgroup by being able to disengage from pain and/or trauma through avoidance and/or dissociation, a process often associated with pain, trauma, and depression (13, 15, 16, 97). This would result in an increased ability to wander the mind away from painful and traumatic experiences and thus decrease their immediate salience. Avoidant and suppressive responses may alleviate distress in the short term but, in the long term, may become maladaptive and in fact exacerbate emotional behaviors and interfere with one's ability to extinguish fear responses (98), potentially creating neural vulnerability for pain and trauma chronification. Of note, it has also been suggested that within DMN and to PCC connectivity in the context of pain could reflect pain rumination (i.e., perseverative negative thinking about pain) (99), yet this interpretation is less likely since this group did not demonstrate heightened pain or trauma symptoms. We also found that this “medium symptom” subgroup had overlapping functional connections with the “high symptom” subgroup, i.e., the group with the highest clinical impairment. These overlapping functional connections were from insula to ACC. With regard to insula–ACC connectivity, mechanistically, increased connections between limbic sensory cortex (insula) and limbic motor cortex (cingulate) convey stronger affective experience (62, 100) and increased motivation to modulate feeling of pain, and we have previously shown that weaker connections during experimental pain task are associated with increased subjective pain and avoidant brain response in those with combat trauma and PTSD (16). Thus, it is likely, that the increased insula–ACC connectivity in these subgroups underlies avoidant response to pain and trauma, yet in the “medium symptom” subgroup, the avoidant response is effective in reducing subjective experience.

We hypothesized that the model-free approach would identify specific connectivity patterns for increased pain, increased trauma, and their overlap. We did not, however, observe pain-specific vs. trauma-specific circuits in our heterogeneous sample, but the degree of symptoms was driving our subgroup differences. Note that the incidence of meeting the criteria for pain and/or trauma in our sample was not different among the subgroups. This supports the RDoC transdiagnostic notion (29). It is also of note that patients and veterans, in particular, rarely present with a single disorder but often with multiple comorbidities and that treatments tested in uncomplicated patients often fail in clinical settings. This potentially more generalizable approach may direct choices for various non-opioid interventions and need to be tested in the future.

Limitations of the current study include the use of a cross-sectional design, which restricted our ability to draw conclusions as to whether the brain findings represent a vulnerability to, or impact of, chronic pain and/or trauma. It is also important to note that our sample had relatively high levels of education, and although this demographic factor did not differ between groups, its limited range may reduce the generalizability of these findings. Similarly, the proportion of women in this study was low; thus, current findings may not generalize to the female population. In addition, our overall sample size was relatively small but is comparable with other recent studies using rs-fMRI (28). Furthermore, GIMME has been shown to be robust with small sample sizes, which suggests that this is an ideal method for investigations with challenging-to-recruit patient populations. Nonetheless, future work with larger samples will be required to determine if larger samples will also find similar subtypes as those observed in our study and to probe the unique within-subgroup connectivity profiles. In addition, we used S-GIMME, which is a version of uSEM that uses time-series analysis with an SEM framework (36). Although it offers an improvement over simple functional connectivity models (e.g., Pearson's correlation and distance measures) by providing directional and causal relationships between networks (32, 33, 101), it also has several limitations. These include statistical dependence of multiple features, reduced equivalency with other methods, and a lower maximum of allowable features in the model (due to the increased computational load <20 lagged and contemporaneous features are allowed into the model). Nevertheless, this approach, when combined with the RDoC approach, provides a unique opportunity to understand unique functional brain connectivity as it maps to pain and trauma symptoms in heterogeneous samples.

## Conclusions

We report novel evidence of unique functional connectivity patterns associated with pain and trauma symptoms in a sample of veterans. Using an unsupervised approach rather than predefined diagnostic grouping, we identified a unique and overlapping pattern of functional connectivity at rest that represented subjects with differential clinical symptoms subtypes. Our “low symptom” subtype that was characterized by integrated connectivity patterns between the networks, both in the hub (i.e., insula) and pain modulatory networks, may be consistent with the resilient biotype following trauma. Conversely, the “high symptom” subtype was the most interesting candidate as a risk-related biotype. We believe that specific connections or whole-brain patterns are important for determining vulnerability or resilience subtypes and may be used to classify future samples.

## Data Availability Statement

The raw data supporting the conclusions of this article will be made available by the authors, without undue reservation.

## Ethics Statement

The studies involving human participants were reviewed and approved by University of California San Francisco Human Research Protection Program and Veterans Affairs San Francisco Healthcare System Research and Development Committee. The patients/participants provided their written informed consent to participate in this study.

## Author Contributions

IS, ADS, and ANS were all integral in the acquisition of data, provided input for the analysis and interpretation of the data, and gave final approval of the version to be published. All authors contributed to the article and approved the submitted version.

## Funding

This work was supported in part by the United States Department of Veterans Affairs I01-CX-000816, I01-CX- 001652, I01-CX001542, and I01-CX-001762, the National Institute of Arthritis and Musculoskeletal and Skin Diseases of the National Institutes of Health under Award Number U19AR076737, and the Painless Research Foundation.

## Conflict of Interest

The authors declare that the research was conducted in the absence of any commercial or financial relationships that could be construed as a potential conflict of interest.

## Publisher's Note

All claims expressed in this article are solely those of the authors and do not necessarily represent those of their affiliated organizations, or those of the publisher, the editors and the reviewers. Any product that may be evaluated in this article, or claim that may be made by its manufacturer, is not guaranteed or endorsed by the publisher.

## References

[1] Lew HL Otis JD Tun C Kerns RD Clark ME Cifu DX. Prevalence of chronic pain, posttraumatic stress disorder, and persistent postconcussive symptoms in OIF/OEF veterans: polytrauma clinical triad. J Rehabil Res Dev. (2009) 46:697–702. 10.1682/JRRD.2009.01.0006 20104399

[2] Fishbain DA Pulikal A Lewis JE Gao J. Chronic pain types differ in their reported prevalence of Post -Traumatic Stress Disorder (PTSD) and there is consistent evidence that chronic pain is associated with ptsd: an evidence-based structured systematic review. Pain Med. (2017) 18:711–35. 10.1093/pm/pnw065 27188666

[3] Webb EK Huggins AA Belleau EL Taubitz LE Hanson JL deRoon-Cassini TA. Acute posttrauma resting-state functional connectivity of periaqueductal gray prospectively predicts posttraumatic stress disorder symptoms. Biol Psychiatry Cogn Neurosci Neuroimaging. (2020) 5:891–900 10.1016/j.bpsc.2020.03.004 32389746 PMC7483700

[4] Linnemorken LTB Granan LP Reme SE. Prevalence of posttraumatic stress symptoms and associated characteristics among patients with chronic pain conditions in a Norwegian University Hospital outpatient pain clinic. Front Psychol. (2020) 11:749. 10.3389/fpsyg.2020.00749 32431641 PMC7215085

[5] Otis JD Keane TM Kerns RD. An examination of the relationship between chronic pain and post-traumatic stress disorder. J Rehabil Res Dev. (2003) 40:397–405. 10.1682/JRRD.2003.09.0397 15080224

[6] Asmundson GJ Katz J. Understanding the co-occurrence of anxiety disorders and chronic pain: state-of-the-art. Depress Anxiety. (2009) 26:888–901. 10.1002/da.20600 19691031

[7] Higgins DM Kerns RD Brandt CA Haskell SG Bathulapalli H Gilliam W. Persistent pain and comorbidity among operation enduring freedom/operation iraqi freedom/operation new dawn veterans. Pain Med. (2014) 15:782-90. 10.1111/pme.12388 24548466

[8] Seal KH Shi Y Cohen G Cohen BE Maguen S Krebs EE. Association of mental health disorders with prescription opioids and high-risk opioid use in US veterans of Iraq and Afghanistan. JAMA. (2012) 307:940–7. 10.1001/jama.2012.234 22396516

[9] Ng SK Urquhart DM Fitzgerald PB Cicuttini FM Hussain SM Fitzgibbon BM. The relationship between structural and functional brain changes and altered emotion and cognition in chronic low back pain brain changes: a systematic review of MRI and fMRI Studies. Clin J Pain. (2018) 34:237–61. 10.1097/AJP.0000000000000534 28719509

[10] Nutt DJ Malizia AL. Structural and functional brain changes in posttraumatic stress disorder. J Clin Psychiatry. (2004) 65:11−7. 14728092

[11] Moeller-Bertram T Strigo IA Simmons AN Schilling JM Patel P Baker DG. Evidence for acute central sensitization to prolonged experimental pain in posttraumatic stress disorder. Pain Med. (2014) 15:762–71. 10.1111/pme.12424 24738563

[12] Tesarz J Baumeister D Andersen TE Vaegter HB. Pain perception and processing in individuals with posttraumatic stress disorder: a systematic review with meta-analysis. Pain Rep. (2020) 5:e849. 10.1097/PR9.0000000000000849 33490843 PMC7808684

[13] Mickleborough MJ Daniels JK Coupland NJ Kao R Williamson PC Lanius UF. Effects of trauma-related cues on pain processing in posttraumatic stress disorder: an fMRI investigation. J Psychiatry Neurosci. (2011) 36:6–14. 10.1503/jpn.080188 20964954 PMC3004970

[14] Geuze E Westenberg HG Jochims A de Kloet CS Bohus M Vermetten E. Altered pain processing in veterans with posttraumatic stress disorder. Arch Gen Psychiatry. (2007) 64:76–85. 10.1001/archpsyc.64.1.76 17199057

[15] Strigo IA Simmons AN Matthews SC Grimes EM Allard CB Reinhardt LE. Neural correlates of altered pain response in women with posttraumatic stress disorder from intimate partner violence. Biol Psychiatry. (2010) 68:442–50. 10.1016/j.biopsych.2010.03.034 20553750

[16] Strigo IA Spadoni AD Inslicht SS Simmons AN. Repeated exposure to experimental pain differentiates combat traumatic brain injury with and without post-traumatic stress disorder. J Neurotrauma. (2018) 35:297–307. 10.1089/neu.2017.5061 28931334

[17] Elman I Upadhyay J Langleben DD Albanese M Becerra L Borsook D. Reward and aversion processing in patients with post-traumatic stress disorder: functional neuroimaging with visual and thermal stimuli. Transl Psychiatry. (2018) 8:240. 10.1038/s41398-018-0292-6 30389908 PMC6214971

[18] Fox MD Greicius M. Clinical applications of resting state functional connectivity. Front Syst Neurosci. (2010) 4:19. 10.3389/fnsys.2010.00019 20592951 PMC2893721

[19] Apkarian AV Baliki MN Geha PY. Towards a theory of chronic pain. Prog Neurobiol. (2009) 87:81–97. 10.1016/j.pneurobio.2008.09.018 18952143 PMC2650821

[20] Baliki MN Baria AT Apkarian AV. The cortical rhythms of chronic back pain. J Neurosci. (2011) 31:13981–90. 10.1523/JNEUROSCI.1984-11.2011 21957259 PMC3214084

[21] Wasan AD Loggia ML Chen LQ Napadow V Kong J Gollub RL. Neural correlates of chronic low back pain measured by arterial spin labeling. Anesthesiology. (2011) 115:364–74 10.1097/ALN.0b013e318220e880. 21720241 10.1097/ALN.0b013e318220e880 PMC3286828

[22] Baliki MN Chialvo DR Geha PY Levy RM Harden RN Parrish TB. Chronic pain and the emotional brain: specific brain activity associated with spontaneous fluctuations of intensity of chronic back pain. J Neurosci. (2006) 26:12165–73. 10.1523/JNEUROSCI.3576-06.2006 17122041 PMC4177069

[23] Tagliazucchi E Balenzuela P Fraiman D Chialvo DR. Brain resting state is disrupted in chronic back pain patients. Neurosci Lett. (2010) 485:26–31. 10.1016/j.neulet.2010.08.053 20800649 PMC2954131

[24] Hashmi JA Baliki MN Huang L Baria AT Torbey S Hermann KM. Shape shifting pain: chronification of back pain shifts brain representation from nociceptive to emotional circuits. Brain. (2013) 136:2751–68. 10.1093/brain/awt211 23983029 PMC3754458

[25] Baliki MN Petre B Torbey S Herrmann KM Huang L Schnitzer TJ. Corticostriatal functional connectivity predicts transition to chronic back pain. Nat Neurosci. (2012) 15:1117–9. 10.1038/nn.3153 22751038 PMC3411898

[26] Ceko M Shir Y Ouellet JA Ware MA Stone LS Seminowicz DA. Partial recovery of abnormal insula and dorsolateral prefrontal connectivity to cognitive networks in chronic low back pain after treatment. Hum Brain Mapp. (2015) 36:2075–92. 10.1002/hbm.22757 25648842 PMC6869701

[27] Akiki TJ Averill CL Abdallah CG. A network-based neurobiological model of PTSD: evidence from structural and functional neuroimaging studies. Curr Psychiatry Rep. (2017) 19:81. 10.1007/s11920-017-0840-4 28924828 PMC5960989

[28] Stout DM Harlé KM Norman SB Simmons AN Spadoni AD. Resting-state connectivity subtype of comorbid PTSD and alcohol use disorder moderates improvement from integrated prolonged exposure therapy in Veterans. Psychol Med. (2021) 30:1-10. 10.1017/S0033291721001513 33926595 PMC10880798

[29] Braun U Schaefer A Betzel RF Tost H Meyer-Lindenberg A Bassett DS. A Meyer-Lindenberg, and DS Bassett From Maps to Multi-dimensional Network Mechanisms of Mental Disorders. Neuron. (2018) 97:14–31. 10.1016/j.neuron.2017.11.007 29301099 PMC5757246

[30] Drysdale AT Grosenick L Downar J Dunlop K Mansouri F Meng Y. Resting-state connectivity biomarkers define neurophysiological subtypes of depression. Nat Med. (2017) 23:28–38. 10.1038/nm.4246 27918562 PMC5624035

[31] Zhang X Braun U Tost H Bassett DS. Data-driven approaches to neuroimaging analysis to enhance psychiatric diagnosis and therapy. Biol Psychiatry Cogn Neurosci Neuroimaging. (2020) 5:780–90. 10.1016/j.bpsc.2019.12.015 32127291

[32] Friston KJ. Functional and effective connectivity: a review. Brain Connect. (2011) 1:13–36. 10.1089/brain.2011.0008 22432952

[33] Reid AT Headley DB Mill RD Sanchez-Romero R Uddin LQ Marinazzo D. Advancing functional connectivity research from association to causation. Nat Neurosci. (2019) 22:1751–60. 10.1038/s41593-019-0510-4 31611705 PMC7289187

[34] Gates KM Lane ST Varangis E Giovanello K Guskiewicz K. Unsupervised classification during time-series model building. Multivariate Behav Res. (2017) 52:129–48. 10.1080/00273171.2016.1256187 27925768 PMC8549846

[35] Gates KM Molenaar PC. Group search algorithm recovers effective connectivity maps for individuals in homogeneous and heterogeneous samples. Neuroimage. (2012) 63:310–9. 10.1016/j.neuroimage.2012.06.026 22732562

[36] Gates KM Molenaar PC Hillary FG Ram N Rovine MJ. Automatic search for fMRI connectivity mapping: an alternative to Granger causality testing using formal equivalences among SEM path modeling, VAR, and unified SEM. Neuroimage. (2010) 50:1118–25. 10.1016/j.neuroimage.2009.12.117 20060050

[37] Mumford JA Ramsey JD. Bayesian networks for fMRI: a primer. Neuroimage. (2014) 86:573–82. 10.1016/j.neuroimage.2013.10.020 24140939

[38] Sheehan D Janavs J Baker R Harnett-Sheehan K Knapp E Sheehan M. Mini International Neuropsychiatric Interview. Tampa: University of South Florida (1994).

[39] Cleeland CS Ryan K. The brief pain inventory. Pain Res Group. (1991) 28:143–7.

[40] Weathers F. B. Litz T. Keane P. Palmieri B. Marx P. Schnurr. The PTSD Checklist for DSM-5 (PCL-5). Scale Available National Center PTSD. (2013) 10

[41] Beck AT Steer RA Brown GK. Manual for the Beck Depression Inventory-II. San Antonio, TX: Psychological Corporation (1996). p. 1–82.

[42] Sullivan M Bishop S Pivik J. The pain catastrophizing scale: develompent and validation. Psychol Assess. (1995) 7:524–32. 10.1037/1040-3590.7.4.52428616005

[43] McCracken LM Dhingra L. A short version of the Pain Anxiety Symptoms Scale (PASS-20): preliminary development and validity. Pain Res Manag. (2002) 7:45–50. 10.1155/2002/517163 16231066

[44] McCarthy DM Pedersen SL Thompsen DM Leuty ME. Clinical evaluation of a measure to assess combat exposure. Psychol Assess J Consult Clin Psychol. (1989) 1:53–5. 10.1037/1040-3590.1.1.53

[45] Protocol ATI. Trauma-Informed Care in Behavioral Health Services. Rockville, USA: Substance Abuse and Mental Health Services Administration (2014) 24901203

[46] Treede RD Rief W Barke A Aziz Q Bennett MI Benoliel R. A classification of chronic pain for ICD-11. Pain. (2015) 156:1003–7. 10.1097/j.pain.0000000000000160 25844555 PMC4450869

[47] Esteban O Markiewicz CJ Blair RW Moodie CA Isik AI Erramuzpe A. fMRIPrep: a robust preprocessing pipeline for functional MRI. Nat Meth. (2019) 16:111–6. 10.1038/s41592-018-0235-4 30532080 PMC6319393

[48] Gorgolewski KJ Esteban O Markiewicz CJ Ziegler E Ellis DG Notter MP. Nipype. Software, Zenodo (2018).

[49] Gorgolewski K Burns CD Madison C Clark D Halchenko YO Waskom ML. Nipype: a flexible, lightweight and extensible neuroimaging data processing framework in python. Front Neuroinform. (2011) 5:13. 10.3389/fninf.2011.00013 21897815 PMC3159964

[50] Faillenot I Heckemann RA Frot M Hammers A. Macroanatomy and 3D probabilistic atlas of the human insula. Neuroimage. (2017) 150:88–98. 10.1016/j.neuroimage.2017.01.073 28179166

[51] Hammers A Allom R Koepp MJ Free SL Myers R Lemieux L. Three-dimensional maximum probability atlas of the human brain, with particular reference to the temporal lobe. Hum Brain Mapp. (2003) 19:224–47. 10.1002/hbm.10123 12874777 PMC6871794

[52] Craig AD. How do you feel? Interoception: the sense of the physiological condition of the body. NatRevNeurosci. (2002) 3:655–66. 10.1038/nrn894 12154366

[53] Fields HL. How expectations influence pain. Pain. (2018) 159: S3–10. 10.1097/j.pain.0000000000001272 30113941

[54] Baliki MN Mansour AR Baria AT Apkarian AV. Functional reorganization of the default mode network across chronic pain conditions. PLoS ONE. (2014) 9:e106133. 10.1371/journal.pone.0106133 25180885 PMC4152156

[55] Stevens JS Harnett NG Lebois LAM van Rooij SJH Ely TD Roeckner A. Brain-based biotypes of psychiatric vulnerability in the acute aftermath of trauma. Am J Psychiatry. (2021) 178:1037–49. 10.1176/appi.ajp.2021.20101526 34645277 PMC9069566

[56] Gates KM Henry T Steinley D Fair DA. A Monte Carlo evaluation of weighted community detection algorithms. Front Neuroinform. (2016) 10:45. 10.3389/fninf.2016.00045 27891087 PMC5102890

[57] Orman GK Labatut V. A comparison of community detection algorithms on artificial networks. In: International Conference on Discovery Science. Berlin: Springer (2009).

[58] Price RB Gates K Kraynak TE Thase ME Siegle GJ. Data-driven subgroups in depression derived from directed functional connectivity paths at rest. Neuropsychopharmacology. (2017) 42:2623–32. 10.1038/npp.2017.9 28497802 PMC5686504

[59] Price RB Lane S Gates K Kraynak TE Horner MS Thase ME. Parsing heterogeneity in the brain connectivity of depressed and healthy adults during positive mood. Biol Psychiatry. (2017) 81:347–57. 10.1016/j.biopsych.2016.06.023 27712830 PMC5215983

[60] JASP Team,. JASP (Version 0.16.2). (2022). Available online at: https://jasp-stats.org/faq/how-do-i-cite-jasp/

[61] Craig AD. How do you feel [mdash] now? The anterior insula and human awareness. Nat Rev Neurosci. (2009) 10:59–70. 10.1038/nrn2555 19096369

[62] Craig AD. How do You Feel?: An Interoceptive Moment With Your Neurobiological Self. USA: Princeton University Press (2015). 10.1515/9781400852727

[63] Craig AD. Referee Report For: The dorsal posterior insula is not an island in pain but subserves a fundamental role - Response to: “Evidence against pain specificity in the dorsal posterior insula” by Davis et al. F1000Res. (2015) 4:1207. 10.12688/f1000research.7287.1 26834997 PMC4706052

[64] Segerdahl AR Mezue M Okell TW Farrar JT Tracey I. The dorsal posterior insula subserves a fundamental role in human pain. Nat Neurosci. (2015) 18:499–500. 10.1038/nn.3969 25751532 PMC6783299

[65] Menon V Uddin LQ. Saliency, switching, attention and control: a network model of insula function. Brain Struct Funct. (2010) 214:655–67. 10.1007/s00429-010-0262-0 20512370 PMC2899886

[66] Simmons AN Norman SB Spadoni AD Strigo IA. Neurosubstrates of remission following prolonged exposure therapy in veterans with posttraumatic stress disorder. Psychother Psychosom. (2013) 82:382–9. 10.1159/000348867 24061484

[67] Strigo IA Matthews SC Simmons AN. Decreased frontal regulation during pain anticipation in unmedicated subjects with major depressive disorder. Transl Psychiatry. (2013) 3:e239. 10.1038/tp.2013.15 23481626 PMC3625914

[68] Simmons A Strigo IA Matthews SC Paulus MP Stein MB. Initial evidence of a failure to activate right anterior insula during affective set shifting in posttraumatic stress disorder. Psychosom Med. (2009) 71:373–7. 10.1097/PSY.0b013e3181a56ed8 19398499 PMC2888032

[69] Strigo IA Matthews SC Simmons AN Oberndorfer T Klabunde M Reinhardt LE. Altered insula activation during pain anticipation in individuals recovered from anorexia nervosa: Evidence of interoceptive dysregulation. Int J Eat Disord. (2013) 46:23–33. 10.1002/eat.22045 22836447 PMC3507323

[70] Oberndorfer T Simmons A McCurdy D Strigo I Matthews S Yang T. Greater anterior insula activation during anticipation of food images in women recovered from anorexia nervosa versus controls. Psychiatry Res Neuroimag. (2013) 214:132–41. 10.1016/j.pscychresns.2013.06.010 23993362 PMC3880160

[71] Strigo IA Matthews SC Simmons AN. Right anterior insula hypoactivity during anticipation of homeostatic shifts in major depressive disorder. Psychosom Med. (2010) 72:316–23. 10.1097/PSY.0b013e3181d07873 20100882 PMC2884370

[72] Mogenson GJ Jones DL Yim CY. From motivation to action: functional interface between the limbic system and the motor system. Prog Neurobiol. (1980) 14:69–97. 10.1016/0301-0082(80)90018-0 6999537

[73] Roesch MR Singh T Brown PL Mullins SE Schoenbaum G. Ventral striatal neurons encode the value of the chosen action in rats deciding between differently delayed or sized rewards. J Neurosci. (2009) 29:13365–76 10.1523/JNEUROSCI.2572-09.2009 19846724 PMC2788608

[74] Cauda F Cavanna AE D'agata F Sacco K Duca S Geminiani GC. Functional connectivity and coactivation of the nucleus accumbens: a combined functional connectivity and structure-based meta-analysis. J Cogn Neurosci. (2011) 23:2864–77. 10.1162/jocn.2011.21624 21265603

[75] Kadlec M Tosun D Strigo I. BOLD decoding of individual pain anticipation biases during uncertainty. bioRxiv. (2019) 675645. 10.1101/675645

[76] Baliki MN Geha PY Fields HL Apkarian AV. Predicting value of pain and analgesia: nucleus accumbens response to noxious stimuli changes in the presence of chronic pain. Neuron. (2010) 66:149–60. 10.1016/j.neuron.2010.03.002 20399736 PMC2873199

[77] Schwartz N Miller C Fields HL. Cortico-accumbens regulation of approach-avoidance behavior is modified by experience and chronic pain. Cell Rep. (2017) 19:1522–31. 10.1016/j.celrep.2017.04.073 28538173

[78] Ungless MA Magill PJ Bolam JP. Uniform inhibition of dopamine neurons in the ventral tegmental area by aversive stimuli. Science. (2004) 303:2040–2. 10.1126/science.1093360 15044807

[79] Seymour B O'Doherty JP Dayan P Koltzenburg M Jones AK Dolan RJ. Temporal difference models describe higher-order learning in humans. Nature. (2004) 429:664–7. 10.1038/nature02581 15190354

[80] Zubieta JK Bueller JA Jackson LR Scott DJ Xu Y Koeppe RA. Placebo effects mediated by endogenous opioid activity on mu-opioid receptors. J Neurosci. (2005) 25:7754–62. 10.1523/JNEUROSCI.0439-05.2005 16120776 PMC6725254

[81] Eippert F Bingel U Schoell ED Yacubian J Klinger R Lorenz J. Activation of the opioidergic descending pain control system underlies placebo analgesia. Neuron. (2009) 63:533–43. 10.1016/j.neuron.2009.07.014 19709634

[82] Baliki MN Schnitzer TJ Bauer WR Apkarian AV. Brain morphological signatures for chronic pain. PLoS ONE. (2011) 6:e26010. 10.1371/journal.pone.0026010 22022493 PMC3192794

[83] Vachon-Presseau E Tétreault P Petre B Huang L Berger SE Torbey S. Corticolimbic anatomical characteristics predetermine risk for chronic pain. Brain. (2016) 139:1958–70. 10.1093/brain/aww100 27190016 PMC4939699

[84] Makary MM Polosecki P Cecchi GA DeAraujo IE Barron DS Constable TR. Loss of nucleus accumbens low-frequency fluctuations is a signature of chronic pain. Proc Natl Acad Sci USA. (2020) 117:10015–23. 10.1073/pnas.1918682117 32312809 PMC7211984

[85] Benarroch EE. Involvement of the nucleus accumbens and dopamine system in chronic pain. Neurology. (2016) 87:1720–6. 10.1212/WNL.0000000000003243 27655737

[86] Apkarian AV Sosa Y Krauss BR Thomas PS Fredrickson BE Levy RE. Chronic pain patients are impaired on an emotional decision-making task. Pain. (2004) 108:129–36. 10.1016/j.pain.2003.12.015 15109516

[87] Geha P deAraujo I Green B Small DM. Decreased food pleasure and disrupted satiety signals in chronic low back pain. Pain. (2014) 155:712–22. 10.1016/j.pain.2013.12.027 24384160

[88] Schwartz N Temkin P Jurado S Lim BK Heifets BD Polepalli JS. Chronic pain Decreased motivation during chronic pain requires long-term depression in the nucleus accumbens. Science. (2014) 345:535–42. 10.1126/science.1253994 25082697 PMC4219555

[89] Ramirez F Moscarello JM LeDoux JE Sears RM. Active avoidance requires a serial basal amygdala to nucleus accumbens shell circuit. J Neurosci. (2015) 35:3470–7. 10.1523/JNEUROSCI.1331-14.2015 25716846 PMC4339356

[90] De Groote A de Kerchove d'Exaerde A. Thalamo-Nucleus Accumbens Projections in Motivated Behaviors and Addiction. Front Syst Neurosci. (2021) 15:711350. 10.3389/fnsys.2021.711350 34335197 PMC8322971

[91] Leech R Sharp DJ. The role of the posterior cingulate cortex in cognition and disease. Brain. (2014) 137:12–32. 10.1093/brain/awt162 23869106 PMC3891440

[92] Chase HW Auerbach RP Brent DA Posner J Weissman MM Talati A. Dissociating default mode network resting state markers of suicide from familial risk factors for depression. Neuropsychopharmacology. (2021) 46:1830–8. 10.1038/s41386-021-01022-5 34059799 PMC8358011

[93] Kucyi A Salomons TV Davis KD. Mind wandering away from pain dynamically engages antinociceptive and default mode brain networks. Proc Natl Acad Sci USA. (2013) 110:18692–7. 10.1073/pnas.1312902110 24167282 PMC3832014

[94] Mason MF Norton MI Van Horn JD Wegner DM Grafton ST Macrae CN. Wandering minds: the default network and stimulus-independent thought. Science. (2007) 315:393–5. 10.1126/science.1131295 17234951 PMC1821121

[95] Kucyi A Davis KD. The dynamic pain connectome. Trends Neurosci. (2015) 38:86–95. 10.1016/j.tins.2014.11.006 25541287

[96] Wiech K Ploner M Tracey I. Neurocognitive aspects of pain perception. Trends Cogn Sci. (2008) 12:306–13. 10.1016/j.tics.2008.05.005 18606561

[97] Defrin R Schreiber S Ginzburg K. Paradoxical pain perception in posttraumatic stress disorder: the unique role of anxiety and dissociation. J Pain. (2015) 16:961–70. 10.1016/j.jpain.2015.06.010 26168878

[98] Foa EB Kozak MJ. Emotional processing of fear: exposure to corrective information. Psychol Bull. (1986) 99:20–35. 10.1037/0033-2909.99.1.20 2871574

[99] Kucyi A Moayedi M Weissman-Fogel I Goldberg MB Freeman BV Tenenbaum HC. Enhanced medial prefrontal-default mode network functional connectivity in chronic pain and its association with pain rumination. J Neurosci. (2014) 34:3969–75. 10.1523/JNEUROSCI.5055-13.2014 24623774 PMC6705280

[100] Touroutoglou A Hollenbeck M Dickerson BC Feldman Barrett L. Dissociable large-scale networks anchored in the right anterior insula subserve affective experience and attention. Neuroimage. (2012) 60:1947–58. 10.1016/j.neuroimage.2012.02.012 22361166 PMC3345941

[101] Stephan KE Friston KJ. Analyzing effective connectivity with functional magnetic resonance imaging. Wiley Interdiscip Rev Cogn Sci. (2010)1:446–59. 10.1002/wcs.58 21209846 PMC3013343

